# Supplementary material for: Oral Formulation Based on Irbesartan Nanocrystals Improve Drug Solubility, Absorbability, and Efficacy
Source: Pharmaceutics. 2022 Feb 10;14(2):387. doi: 10.3390/pharmaceutics14020387 (PMC8875686; doi:10.3390/pharmaceutics14020387)
Supplement: Supplementary file 1 [file pharmaceutics-14-00387-s001.zip › pharmaceutics-1569048-supplementary.pdf]

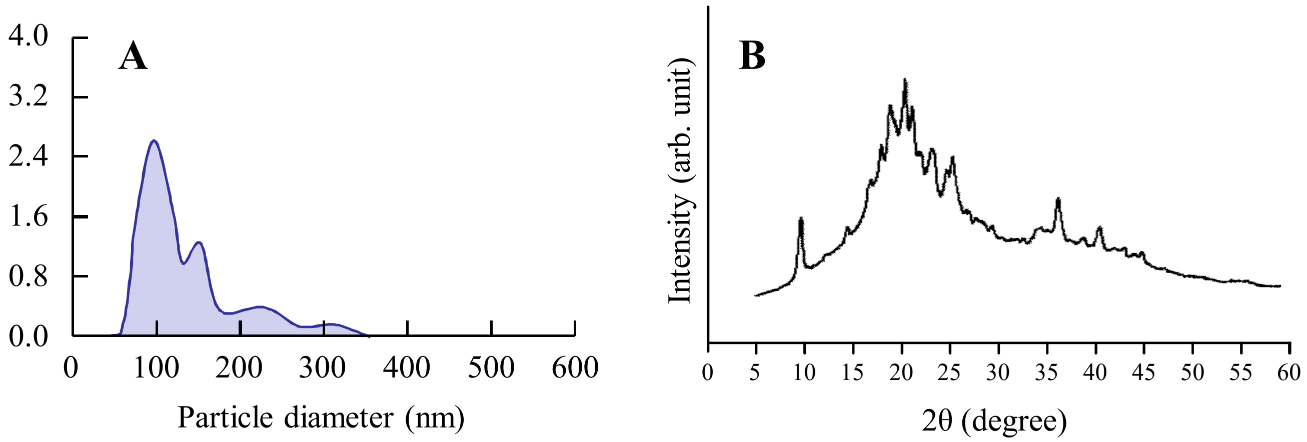

Supplemental data 1 (Figure S1). Characterization of the 3-month-stored IRB-NP tablets. (A) Particle size frequencies of IRB in the redispersions of 3-month-stored IRB-NP tablets. (B) Powder X-ray diffraction patterns of 3-month-stored IRB-NP tablets.
